# Preventing chemotherapy-induced diarrhea and microbiota imbalances with prebiotics and probiotics in breast cancer treatment: A case report

**DOI:** 10.1080/29933935.2024.2379475

**Published:** 2024-08-13

**Authors:** Patricia Kaufman, K. Erin O’Meara, Jason Hawrelak

**Affiliations:** a College of Nutrition, Sonoran University of Health Sciences, Tempe, AZ, USA; b Integrative Health, American College of Healthcare Sciences, Portland, OR, USA; c Independent Researcher, Minneapolis, MN, USA; d School of Pharmacy and Pharmacology, University of Tasmania, Hobart, TAS, Australia; e Human Nutrition and Functional Medicine, University of Western States, Portland, OR, USA; f The Microbiome Restoration Center, Ocean Shores, NSW, Australia

**Keywords:** Gut bacteria, chemotherapy adverse effects, microbiome, probiotics, prebiotics

## Abstract

Breast cancer (BC) is the second most common cancer in women in the United States. Of those diagnosed, 40–80% will undergo chemotherapy. Adverse effects of chemotherapy are chemotherapy-induced diarrhea (CID) and gut microbiota dysregulation. CID can lead to dehydration, metabolic acidosis, malnutrition, and gut dysbiosis. Antidiarrheal medications are the standard treatment of care; however, this has been shown to further contribute to gut dysbiosis, is not always effective in controlling diarrhea, and can lead to rebound constipation with the potential of pathogenic bacterial overgrowth. In this case report, we describe the experience of a patient-centered, personalized intervention with pre- and probiotics to preserve the microbiota and prevent CID. A 57-year-old postmenopausal female with BC undergoing adriamycin-cyclophosphamide (AC) and taxol-cyclophosphamide (TC) chemotherapies for invasive ductal carcinoma under the care of a cancer team wanted to refrain from using loperamide and instead use nutritional interventions and supplementation for preventing CID and maintaining gut health. This case report is a narrative report of the observed outcomes of one patient with BC after taking specific prebiotics and probiotics. The outcomes included the prevention of CID and other gastrointestinal adverse effects, and maintaining microbiota alpha-diversity, butyrate producing genera, and *Bifidobacterium* populations while inhibiting the overgrowth of *Proteobacteria* pathogenic bacteria.

## Introduction

Breast cancer (BC) is the number one cancer in the United States for women after skin cancer. In 2022, it is estimated that there will be 290,560 incidences of breast cancer in the United States.^1,2^ Of the women diagnosed with BC, 40–80% will undergo chemotherapy, and 52% will receive radiation as part of breast-conserving surgery.^3^ Chemotherapy-induced diarrhea (CID) is one of the most common and serious adverse drug effects and afflicts between 50% and 80% of women undergoing chemotherapy.^4^ CID can lead to dehydration, electrolyte imbalance leading to metabolic acidosis, malnutrition, gut dysbiosis, and decreased quality of life.^5^ Chemotherapy and radiation treatments have been shown to disrupt the microbiota balance by decreasing bacterial richness and bifidobacteria populations (beneficial bacteria) while increasing pathogenic bacteria. These changes in the gut microbiota can be compounding factors of CID.^6–11^

Treatment and hospitalization due to CID can be dose-limiting and lead to treatment delays, indirectly affecting recurrence, outcomes, and mortality.^4,5,12^ The standard of care for CID is an antidiarrheal medication, such as loperamide.^10^ However, loperamide can cause constipation by slowing motility, which increases the risk of an overgrowth of pathogenic bacteria.^13^

Limited data is available evaluating the use of the combination of prebiotics and probiotics for managing CID and chemotherapy-induced microbiota disturbances. The purpose of this case report is to provide a narrative of the observed findings of one patient for managing CID and preserving bacterial diversity and microbiota balance during and after chemoradiotherapy by using a personalized, specific prebiotic and probiotic protocol.

The CARE reporting guidelines and checklist, an international standard for case reports, were utilized to construct the reporting of these findings to assure high quality, transparency, consistency, and completeness.^14^

## 16S rRNA sequencing and gut bacteria testing products

The gut microbiota of the client was assessed by utilizing 16S rRNA technology. This approach uses 16S rRNA amplicon sequencing of gut microbiota and has a high confidence (96%) at the genus level for the identification of bacterial populations.^15^ Sampling kits were obtained from Ombré Lab, a commercially available product not evaluated by the Food and Drug Administration (FDA). The product is not intended to diagnose, treat, cure, or prevent disease.

Fecal swab samples were collected by the patient, mailed to and processed by Laragen Sequencing and Genotyping (Culver City, CA, USA) (April 2021, March 2022) and Eurofins Clinical Enterprise (Framingham, MA, USA) (August 2022), where DNA was isolated and sequencing was performed. The V4 region was amplified and sequenced using Earth Microbiome 16S protocol^16^ (Laragen) and Zymo Quick NGS protocol^17^ (Eurofins) library prep kits, respectively. Primers used by labs were 515F/806 R, and 341f − 806 r, respectively.

Sequencing was done on the Illumina MiSeq (Hayward, CA, USA), and read counts were 20,000–100,000 and data were processed without normalization/rarefaction. Laragen and Eurofin supplied FASTQ files, which were run through both Green Genes^18^ and RDP^19^ matching engines via Biomesight, to verify bacterial populations. The FASTQ files were submitted to the National Center for Biotechnology Information (NCBI). The accession number is SRP48933. All data presented in this case report were obtained from the RDP interpretation.

### Prebiotics and Probiotics Products

The prebiotics and probiotics used by the client are third-party tested supplements, utilizing Good Manufacturing Practices (GMP), ensuring standardization and regulations for supplement safety and manufacturing food safety.^20,21,22^ The brands used include Metagenics (Aliso Viejo, CA, USA), Culturelle (Amerifit, Cromwell, CT, USA), Florastor (Biocodex, Beauvais, France), and Healthy Origins Healthy Fiber (the brand of SunFiber, Pittsburgh, PA, USA). Metagenics supplements are certified by NSF, TGA, and USP. Culturelle follows the GMP set forth by the FDA. Florastor is NSF certified. Healthy Origins is certified by cGMP.

## Case narrative

**Figure 1.**
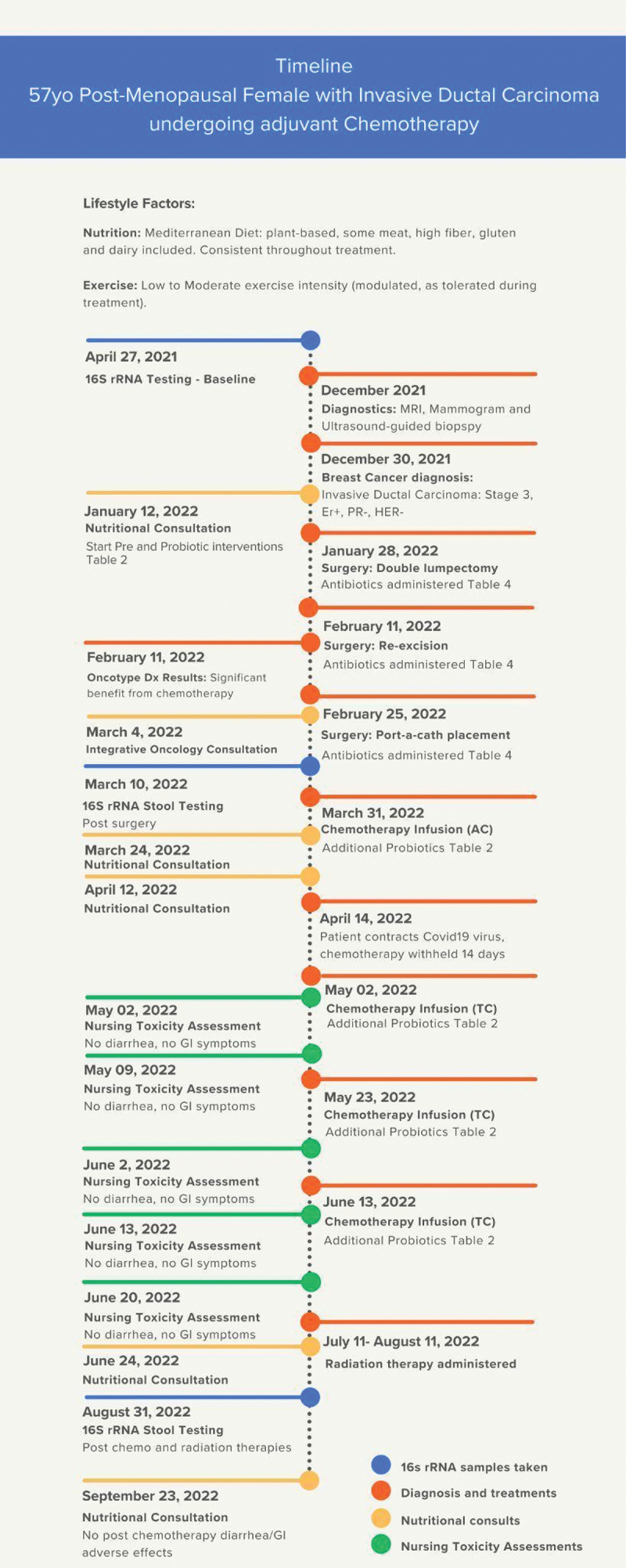
Case timeline.

A 57-year-old postmenopausal woman with a past history of irritable bowel syndrome (IBS) was diagnosed on December 30, 2021, with Breast Cancer (BC); early-stage invasive ductal carcinoma, Stage 3a, ER positive, PR negative, HER2 negative, 1–3 node positive. An Oncotype Dx (tumor genomic testing) score of 31 showed a significant benefit from chemotherapy, and the patient decided to proceed (Figure 1). Prior to diagnosis, the patient had successfully managed her IBS over several years, and therefore one of her primary goals was to avoid both acute and chronic gastrointestinal side effects, including a flare of IBS, and to maintain gut microbiota balance.

Upon diagnosis, the patient consulted with her integrative primary care provider, nutritionists, naturopathic doctors, and an integrative oncologist to formulate a personalized, safe, and effective nutrition and lifestyle plan to create health and to attempt to avoid the side effects of breast cancer treatment. She consulted with this nutrition care team to support the gut microbiota and reduce the risk of GI adverse effects from surgery, chemo and radiation therapies. The complete timeline is shown in Figure 1.

### Initial nutritional consultation: January 12, 2022

The patient completed nutritional intake forms that were utilized to assess nutritional status, exercise, and sleep quality. Results from a 16s rRNA stool test, taken on April 27, 2021, were used as a baseline for her gut bacteria diversity and microbiota balance (Table 1). The results include *Proteobacteria* abundance at 1.604%, *Bifidobacteria* at 0.488%, the nine butyrate producers analyzed at 34.09%, and alpha-diversity at 3.26% (See the Microbiota Abundance and Diversity section for more details on how the microbiota from the stool tests was assessed.). Based on this initial assessment, the patient’s history of irritable bowel syndrome (IBS), the increased stress from the cancer diagnosis, and the upcoming surgery requiring antibiotics, the patient was recommended a personalized prebiotic and probiotic protocol (Table 2).

**Table 1.** Key microbiota abundance and diversity.

Relative Abundance of Microbiota^a^	Date of 16S rRNA Sampling		
Key Butyrate Producing Genera^b^	April 27, 2021	March 10, 2022	August 31, 2022
*Faecalibacterium*	20.158%	10.088%	15.797%
*Eubacterium (E. rectale only)*	0.779%	0.259%	1.872%
*Roseburia*	4.955%	2.848%	9.326%
*Anaerostipes*	2.369%	0.613%	0.509%
*Clostridium*	2.415%	0.425%	0.867%
*Ruminococcus*	1.464%	0.593%	5.703%
*Coprococcus*	1.102%	0.487%	0.502%
*Butyrivibrio*	0.007%	0%	0.035%
*Butyricicoccus*	0.836%	0.083%	0.066%
Total	34.085%	15.396%	34.677%
Change compared to previous test^c^		−54.83%	125.23%
*Proteobacteria*	1.604%	2.319%	0.521%
Change compared to previous test		44.58%	−77.53%
*Bifidobacterium*	0.488%	2.719%	1.125%
Change compared to previous test		457.17%	−58.63%
Shannon alpha diversity index	3.26%	2.58%	2.99%
Change compared to previous test		−20.86%	15.89%

Note: ^a^The data presented is a percentage of total genera abundance.

^b^Via the acetyl-CoA pathway.

^c^Standard percentage change formula (((value 2 - value 1)/value 1) × 100)) was used to calculate the change from the previous test to the current.

**Table 2.** Prebiotic and probiotic supplements.

		Occasions administered	
Probiotics	Dose	January 12-August 31, 2022	Day prior to and day of infusion*
1. *Lactobacillus rhamnosus*	10 billion CFU	Daily, a.m	
GG^a^			
2. *Saccharomyces cerevisiae var boulardii*	250 mg	Daily, p.m.	
CNCM 1–745 Biocodex^b^			
3. A Probiotic Blend^c^ of: *Bifidobacterium lactis* HN019	4 billion CFU (combined HN019 and HN001)		a.m., p.m
*Lactobacillus rhamnosus* HN001 *Saccharomyces cerevisiae var boulardii* Unspecified strain	5.5 billion CFU		
4. A Probiotic Blend^c^ of: *Lactobacillus acidophilus* NCFM	60 billion CFU		a.m., p.m.
*Bifidobacterium lactis*			
Bi-07			
Prebiotics			
1. Lactulose 10 g/15 ml^d^, Rx (no additives)	15 ml	Daily, p.m.	
2. Partially Hydrolyzed Guar Gum^e^ (PHGG)	7.5 g	Daily, a.m.	

Note:*March 30 and 31, 2022, May 1 and 2, 2022, May 22 and 23, 2022, June 12 and 13, 2022.

Supplement manufacturers.

^a^Amerifit (Cromwell, CT, USA).

^b^Biocodex (Beauvais, France).

^c^Metagenics (Aliso Viejo, CA, USA).

^d^Hi-Tech Pharmacal Co., Inc. (Amityville, NY, USA).

^e^Taiyo International (Pittsburg, PA, USA).

Specific strains of probiotics and particular prebiotics were chosen for their ability to modulate key microbiota players, minimize intestinal dysbiosis, and their role in preventing IBS-related symptoms and antibiotic-associated diarrhea (Table 3). These prebiotics and probiotics were selected based on evidence (Table 3). Partially Hydrolyzed Guar Gum (PHGG), a prebiotic, was chosen to support stool consistency, reduce bloating and gas, and increase key butyrate producing bacteria. Lactulose, also a prebiotic, was selected for its bifidogenic quality, ability to acidify the colon, decrease *Proteobacteria*, and increase key butyrate producing bacteria. *Lactobacillus rhamnosus* GG was chosen for its capability to increase epithelial cell synthesis, gut healing and bacterial diversity, and to decrease the growth of pathogenic bacteria. The team selected *Saccharomyces cerevisiae* var *boulardii* CNCM I-745 for its capacity to decrease antibiotic associated diarrhea, pathogenic bacteria, and inflammation, and for increasing the activity of brush border enzymes.

**Table 3.** Prebiotic and probiotic beneficial outcomes and informed-evidence.

Pre/Probiotic	Outcomes	Evidence
*Lactobacillus rhamnosus* GG	↑ rate of epithelial cell synthesis	61,56
	↑ gut healing	
	↑ bacterial diversity	
	↓ pathogenic bacterial growth	
*Saccharomyces cerevisiae* var *boulardii* CNCM I-745	↓ antibiotic associated diarrhea	67,66
	↑ activity of brush border enzymes	
	↓ pathogenic growth	
	+ antimicrobial	
	+ anti-inflammatory	
*Bifidobacterium lactis* HN019 *Lactobacillus rhamnosus* HN001 *Lactobacillus acidophilus* NCFM *Bifidobacterium lactis* Bi-07	↓ bloating	62,63,64,65
	↓ diarrhea	
	↓ pathogenic bacterial growth	
	↑ bacterial diversity	
Partially hydrolyzed guar gum (PHGG)	↓ diarrhea post radiation	48,50,51,46
	+ stool consistency	
	↓ bloating and gas	
	↑ butyrate producing bacteria	
Lactulose	↑ bifidobacteria	52,57
	↑ butyrate producing bacteria	
	↓ *Proteobacteria*	
	+ acidify the colon	

The patient’s other supplements were reviewed for compatibility with GI function and microbiota goals. It was suggested that the patient conduct another stool test post-surgery to assess any microbiota changes.

Prior to diagnosis, the patient adhered to a plant-focused Mediterranean diet consisting of whole grains, beans, vegetables, fruit, fiber, polyphenol-rich foods, lean meats, and dairy. The nutritional care team advised the patient to slightly adjust her Mediterranean diet by increasing her intake of cruciferous vegetables and adding ground organic flaxseed. The patient’s surgical oncologist recommended increasing dietary protein pre- and post-surgery to facilitate healing.

Prior to diagnosis, the patient was taking levothyroxine 88 mcg. The medication and dose remained the same during treatment.

Prior to diagnosis, the patient was physically active by walking, hiking, cycling, rowing, and gardening. She was encouraged to increase her movement to include an additional 30 minutes of aerobic exercises, such as stationary cycling and rowing, 3–4 times a week, and strength training, 2–3 times per week. The patient received physiotherapy (lymphedema preventative and strength training), acupuncture, shiatsu, chiropractic care, and psychotherapy for stress management and emotional and physical health support.

### Integrative oncology consultation: March 4, 2022

Based on the patient’s Oncotype Dx score, her oncology care team recommended chemotherapy to avoid recurrence. In preparation, the patient’s integrative oncologist, in collaboration with the PharmD, adjusted her nutritional supplements (Table 4) to ensure safety and efficacy and to avoid drug-nutrient interactions with chemotherapy agents and other medications (Table 5). No prebiotic and probiotic protocol change was needed because the integrative oncologist found no potential drug interactions with these products.

**Table 4.** Supplements.

Supplements:	Dose	Supplements taken at diagnosis Jan-Mar 2022	Supplements taken during chemotherapy^a^ Mar-Jun 2022	Supplements taken during radiotherapy^b^ Jul-Aug 2022
Vitamin d3 plus vitamin K	125 mcg 20 mcg	✓	✓	✓
Multivitamin^c^	1 cap daily	✓	✓	✓
Vitamin B6	50 mg		✓	✓
Camellia sinensis (Green tea extract)	200 mg	✓	✓	✓
Zingiber officinale (Ginger)	500 mg		✓	
Magnesium	240 mg	✓	✓	✓
Melatonin	3 mg	✓	✓	✓
EPA and DHA	600 mg 400 mg	✓	✓	✓
Probiotics	See table 2	✓	✓	✓
Prebiotics	See table 2	✓	✓	✓
Vitamin B12	2000 mcg	✓	✓	✓
Vitamin E (mixed tocopherols)	400 mg	✓	✓	
Curcuma longa (Curcumin)	500 mg	✓		✓
Trametes versicolor (Turkey Tail mushroom)	500 mg	✓		✓
Iodine	225 mcg	✓		
CoQ-10	60 mg	✓		
Calcium D-glucarate	500 mg	✓		

Note: ^a^Supplements approved by pharmD & integrative oncologist.

^b^Supplements approved by radiation oncology team.

^c^Pure Encapsulations O.N.E. Multivitamin.

✓ = supplements taken.

**Table 5.** Medications.

Medications: Pre-diagnosis and throughout treatment	Antibiotic administered at each surgery: Jan 22, 2022 Feb 11, 2022 Feb 25, 2022	Chemotherapy 1 cycle: March 31, 2022	Chemotherapy 3 cycles: May 2, 2022 May 23, 2022 June 13, 2022	Chemotherapy support medications*: (steroids, anti-nausea, pain) March 31, 2022- June 13, 2022
Levothyroxine 88mcg/daily	ceFAZolin (ANCEF) IVPB	cyclophosphamide (CYTOXAN) IV	cyclophosphamide (CYTOXAN) IV	fosaprepitant (EMEND)
		DOXOrubicin 2 (ADRIAMYCIN) IV PUSH	DOCEtaxel (TAXOTERE) IV	dexamethasone (DECADRON)
		pegfilgrastim subcutaneous	pegfilgrastim subcutaneous	heparin lock (HEP-LOCK)
				NaCl
				ondansetron (ZOFRAN)
				OLANZapine (ZyPREXA)
				prochlorperazine
				acetaminophen
				oxyCODONE (ROXICODONE)
				loratadine

*Some chemotherapy support medications were “only as needed”.

### Nutritional consultation visit: March 24, 2022

At this point, the patient had undergone three surgeries, two more than expected. These surgeries included a breast-conserving double lumpectomy, a re-excision, and a porto cath placement. To prepare for the upcoming chemotherapies, the nutrition care team met with the patient. The patient had been offered loperamide by her oncology team, to be used in case of CID, however, she opted out to avoid potential side effects.

Based on this consultation, it was recommended she continue with the original regime of prebiotic and probiotics, and add two additional probiotic formulas for support during chemotherapy; only to be used the day before and the day of chemotherapy (Table 2). These additional probiotics were recommended based on evidence that they can mitigate CID and other GI symptoms and assist in preserving the gut microbiota (Table 3). The specific strains were selected: Bifidobacterium lactis HN019 to reduce bloating, Lactobacillus rhamnosus HN001 to reduce diarrhea, Lactobacillus acidophilus NCFM to reduce pathogenic bacterial growth and Bifidobacterium lactis Bi-07 to increase bacterial diversity. The PharmD also approved the new probiotics. The original probiotics were continued daily in order to reduce diarrhea episodes, maintain the integrity of the mucosal lining, increase microbial diversity and limit further pathogenic bacteria overgrowth throughout chemo and radiation therapies. No other changes were made to the patient’s prebiotic and probiotic protocol, nor to her diet and supplements. It was recommended that exercise during the week of chemotherapy treatments be modified to tolerable levels and that she should conduct slow, 30-minute walks daily.

### Nutritional consultation visit: April 12, 2022

Prior to this visit, 16S rRNA results from the March 10th stool sample were available and analyzed for microbiota changes (Table 1). The results include *Proteobacteria* abundance at 2.319% (an increase of 44.58%), *Bifidobacterium* at 2.719% (an increase of 457.17%), the nine butyrate producers analyzed at 15.40% (a decrease of 54.83%), and alpha-diversity at 2.58% (a decrease 20.86% (See the Microbiota Abundance and Diversity section for more details on how the microbiota from the stool tests was assessed.). By this visit, the patient’s first chemotherapy infusion had been administered. She was reassessed for GI function and reported no IBS symptoms, diarrhea, or constipation (Table 6).

**Table 6.** Toxicity assessments and diarrhea grading.

Toxicity Screening Dates					
	May 2, 2022	May 9, 2022	June 2, 2022	June 13, 2022	June 20, 2022
Adverse Event (AE) Diarrhea*					
Grade 1: increase of < 4 stools/day over pre-treatment	✓	✓	✓	✓	✓
Grade 2: increase of 4–6 stools/day, or nocturnal stools	–	–	–	–	–
Grade 3: increase of >/ = 7 stools/day or incontinence; or needs for parenteral support for dehydration	–	–	–	–	–
Grade 4: physiologic consequences requiring intensive care; or hemodynamic collapse	–	–	–	–	–
Grade 5: Death related to adverse event	–	–	–	–	–

Note: * Common Terminology Criteria for Adverse Events (CTCAE) definition of diarrhea: A disorder characterized by an increase in frequency and/or loose or watery bowel movements (National Cancer Institute).^22^

✓ = Patient experience.

Based on this assessment and findings, no changes were made to her prebiotic and probiotic supplement protocols, nor to her supplements and diet at this time, because the team deemed the patient’s care plan to be appropriate to assist at modulating the microbiota to pre-surgical levels and to prevent CID with future chemotherapy infusions.

### Nutritional consultation visit: June 24, 2022

At this visit, 10 days post the final chemotherapy infusion, the patient reported no diarrhea or GI symptoms. Additionally, the patient had been evaluated on May 2, May 9, June 2, June 13 and June 20, 2022, by her oncology care team, via the Nursing Toxicity Assessment, for GI side effects; no diarrhea or GI symptoms were reported (Table 6). Based on this and her upcoming radiotherapy, the only change made to her probiotic and prebiotic protocol was to eliminate those formulas used in the two days before chemotherapy infusions (Table 2). Otherwise, her probiotic and prebiotic protocol, and diet remained the same.

Slight changes were made by her oncology team to add back in supplements that were avoided during chemotherapy due to contraindications and to exclude those contraindicated during radiotherapy (Table 4). The nutrition care team recommended another stool test upon completion of radiation therapy.

### Nutritional consultation final visit: September 23, 2022

By this visit, the patient was 17 weeks post-chemotherapy and 6 weeks post-radiation therapy, and had performed another stool test. The 16S rRNA results from the August 31, 2022, stool sample (13 weeks post-chemotherapy, 3 weeks post-radiation therapy) were available and analyzed, and assessed for post-therapies microbiota changes (Table 1). The results include *Proteobacteria* abundance at 0.521% (a decrease of 77.53%), *Bifidobacteria* abundance at 1.125% (a decrease of 58.63%), the nine butyrate producers analyzed at 34.68% (an increase of 125.23%), and alpha-diversity at 2.99% (an increase of 15.89%). (See the Microbiota Abundance and Diversity section for more details on how the microbiota from the stool tests was assessed.).The patient was reassessed for GI symptoms. At this time, she reported no further changes to gastrointestinal function, including diarrhea and IBS. Based on these findings, it was recommended the patient continue taking the prebiotics to positively facilitate modulation of gut microbiota. She was encouraged to continue with the same diet.

## Patient perspective

I have a long history of IBS and worked hard to heal my gut over the last five years. This included diet and lifestyle changes, pre- and probiotics, and somewhat regular gut microbiome testing. My gut has been in a much better place for the last several years; no more IBS, no diarrhea.

When I received the breast cancer diagnosis, and especially the news that I would need chemotherapy, I was really worried it would affect my gut in a bad way and I would develop IBS all over again. Nutritionists specializing in the gut microbiome created a personalized plan to support my gut throughout treatment. Between my medical oncologist, surgical oncologist, primary care doctor, integrative oncologist and PharmD everything was checked for safety.

The type of chemotherapy I received is notorious for causing diarrhea – sometimes lasting for a long time after chemo ends. I was surprised and hugely relieved I had no diarrhea. My gut felt upset for about 24 hours after each infusion, but it was only mild to moderate. During my follow-ups, when the nurses asked me questions about what symptoms I was experiencing, they stared at their screen, ticking boxes. But when they asked about diarrhea, and I said “no”, they would look away from their screen at me in surprise and say “No diarrhea? Everyone gets diarrhea.” I knew then I was extremely fortunate to have the tools to support my gut health throughout treatment.

I had gut microbiota test results from April 2021 to compare to. I tested my microbiome in March 2022 after the three surgeries, and again after chemo and radiation therapies in August 2022. My good bacteria held up despite the chemotherapy.

I am thrilled to have made it through breast cancer treatment without getting the typical diarrhea. And I have no long-term adverse effects on my GI health – and have had no recurrence of my IBS. I am so grateful to my nutritional care team for their amazing help. I know how important gut health is to overall health, and as I go forward, trying to avoid recurrence, I have some peace of mind, knowing I did everything I could to support that particular aspect of my health.

## Microbiota abundance and diversity

**Figure 2.**
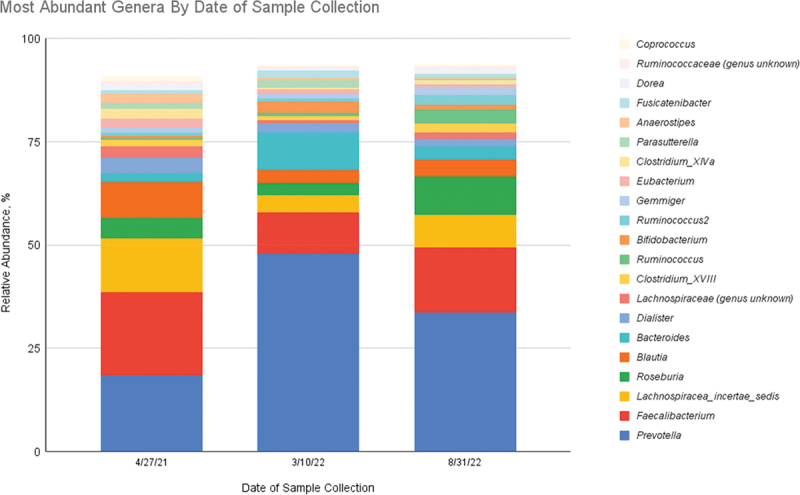
Most abundant genera by date of sample collection.

Gut microbiota were sequenced via 16S rRNA gene-based amplicon to identify taxonomy and observe changes in abundance of several key microbiota prior to breast cancer diagnosis (April 2021, baseline sample), after the introduction of nutritional strategies and post-surgically (March 2022 sample), 13 weeks post-chemotherapy, and 3 weeks post-radiation therapy (August 2022 sample). Among all three samples, the most abundant genera included *Prevotella* (April 2021, 18.5%; March 2022, 47.87%; and August 2022, 33.63%), *Faecalibacterium* (April 2021, 20.16%; March 2022, 10.09%; and August 2022 15.8%), *Lachnospiracea_incertae_sedis (Eubacterium) (April 2021, 12.98%; March 2022, 4.19%; and August 2022 7.84%)*, *Roseburia (April 2021, 4.96%; March 2022, 2.85;, and August 2022, 9.33%)*, *Blautia* (April 2021, 8.7%; March 2022, 3.24%; and August 2022, 4.18%), *Bacteroides* (April 2021, 2.07%; March 2022, 8.97%; and August 2022, 3.07%), *Dialister* (April 2021, 3.7%; March 2022, 2.26%; and August 2022, 1.77%), and *Lachnospiraceae (genus unknown) (April 2021, 2.75% March 2022, 0.82%; and August 2022,1.62%)* (Figure 2).

The nine genera highlighted by Vital et al.^23^ were used as the primary producers of butyrate via the acetyl-CoA pathway: *Faecalibacterium*, *Eubacterium*, *Roseburia*, *Anaerostipes*, *Clostridium*, *Ruminococcus*, *Coprococcus*, *Butyrivibrio*, *Butyricicoccus*. The abundance of these microbiota was analyzed as a potential indicator of butyrate presence. No measurements of butyric acid were performed. These nine butyrate producers decreased by 54% (34.09% to 15.40%) after three surgeries (March 2022 sample) and increased by 125.23% (15.40% to 34.68%) post chemo and radiation therapies (August 2022 sample) (Table 1).

*Proteobacteria* increased 69.51% (1.604% to 2.319%) in the March 2022 sample and decreased again by 77.53% (2.319% to 0.521%) in the August 2022 sample (Table 1). The negative changes on the March 10, 2022, 16S rRNA stool test might be due to the three surgeries (January 28, 2022, February 11, 2022, and February 25, 2022), which included antibiotics (Table 5). Nalluri-Butz and colleagues^24^ showed the expansion of *Proteobacteria* after colorectal resectional and nonresectional surgeries. Additionally, Fan and researchers^25^ illustrated a significant increase in *Proteobacteria* post-surgery in patients with breast cancer surgery. *Bifidobacterium* increased by 457.17% (.488% to 2.719%) in the March 2022 sample and decreased by 58.63% (2.719% to 1.125%) in the August 2022 sample. After completion of cancer treatments, *Bifidobacterium* was more abundant (1.125%) than it was at baseline (0.488%) (Table 1).

Alpha diversity was calculated using the Shannon index.^26^ Diversity decreased by 20.86% (3.26% to 2.58%) in March 2022, and increased by 15.89% (2.58% to 2.99%) by August 2022 (Table 1).

After the completion of cancer treatment (chemotherapy, March 31, 2022, to June 13, 2022; radiation, July 11, 2022, to August 11, 2022) and prebiotic and probiotic protocol as described in Table 2, the patient’s *Proteobacteria* decreased (1.604% to 0.521%) and *Bifidobacterium* increased (0.488% to 1.125%) compared to baseline (Table 1). The total of the nine butyrate producing genera returned to baseline (34.68%), and alpha-diversity (2.58% to 2.99%) had improved from March 2022, but had not returned to baseline (Table 1).

## Discussion

Chemotherapy and radiation therapies are associated with adverse drug side effects, including diarrhea and an imbalance in the gut microbiota. Approximately 50–80% of women undergoing chemotherapy are afflicted with CID, increasing the risk of dehydration and a decreased quality of life.^4,5,27^ In this case, we observed that using prebiotics and probiotics and adherence to a plant-focused Mediterranean diet the client did not experience CID, as illustrated by the toxicity screenings in her health records. The client was also able to reduce the overgrowth of pathogenic bacteria, maintain alpha-diversity and *Bifidobacterium* populations, and positively modulate the microbiota composition and diversity post-chemo and radiation therapies as shown by 16S rRNA testing results (Table 1).

Loperamide, an opioid, is an antidiarrheal drug used as the standard of care to reduce diarrhea by slowing motility.^4^ Slow colonic motility has been shown to increase the growth of pathogenic bacteria, leading to an imbalance in the microbiota.^13,28,29^ Medications to manage CID do not address the underlying contributing factors of CID, including inflammation of the gut mucosa that further perpetuates diarrhea.^7^ Since loperamide is an opioid, tolerance to the drug may occur, causing reduced inhibition of motility and resulting in increased diarrhea.^30^ The client opted not to receive loperamide because she did not want to risk the side effects of the drug as stated above.

CID is thought to occur due to damage to the gut mucosa, increased motility, increased intestinal permeability, loss of gut barrier protection, changes to the gut microbiota composition, and a decrease in alpha-diversity.^7–10,31,32^ Alpha-diversity is a measurement of the number and quality of the microbiota, often referred to as the richness and evenness of the microbiome.^33^

Chemotherapy and radiation therapies have been shown to cause a reduction in alpha-diversity, SCFA bacteria producers, and *Bifidobacteria*, as well as increase *Proteobacteria* populations.^6–9,11^ Aarnoutse and colleagues^6^ demonstrated in 44 patients receiving chemotherapy for breast cancer a significant inverse relationship between alpha-diversity and diarrhea, as well as an increase in *Proteobacteria*, a phylum containing a number of potentially pathogenic bacteria. Montassier et al.^8^ found similar adverse microbiota changes after chemotherapy, such as a low alpha-diversity and an increase in pathogenic *Proteobacteria*, such as *Klebsiella*. Increased levels of *Proteobacteria* and decreases in *Bifidobacteria* have been shown to be associated with gut inflammation and diarrhea in individuals receiving chemotherapy.^7,8^

The gut microbiota, including metabolites produced by the gut microbiota, has been shown to preserve the gut barrier, modulate immune function and synthesize nutrients. Butyrate is a short-chain fatty acid (SCFA), a signaling molecule, and is the main energy source for colonocytes. SCFAs are produced by several bacterial species and have been shown to reduce colonic inflammation induced by pathogenic bacteria, improve the gut mucosal barrier, and have an anti-inflammatory effect by increasing regulatory T cells and decreasing nuclear NF-κBeta.^33–38^ Additionally, SCFAs have antioxidant properties, aid in increasing water absorption to prevent dehydration and electrolyte imbalances, and improve stool consistency and frequency.^34,39^

Moreover, *Bifidobacteria* have been shown to decrease intestinal inflammation, improve gut integrity and ensure adequate mucus production for mucosal protection against pathogenic bacteria.^8,40^ Prebiotics and probiotics have been shown to influence butyrate producing bacteria and increase the level of *Bifidobacteria*, which can help to minimize the overgrowth of pathogenic bacteria. For these reasons, prebiotics and probiotics were suggested to be included in the patient’s nutritional care plan. An explanation of the role of specific prebiotics and probiotics is described below.

Due to the mechanism of actions of prebiotics and probiotics, they may be a non-pharmaceutical safe and effective method for reducing the risk of CID and improving gut microbiota quality, balance and diversity. Prebiotics are fibers and sugars that are undigestable and not absorbed in the small intestine. Specific beneficial strains of bacteria use prebiotics as a substrate to increase their growth.^41^ Colonic fermentation by some of these beneficial bacteria produces butyrate.^42^ Prebiotic-induced shifts in microbiota composition and a subsequent increase in butyrate production can indirectly affect stool frequency and consistency. PHGG, a soluble fiber, and lactulose, a ketos disaccharide, are associated with increases in the percentage of butyrate-producing bacteria and *Bifidobacteria* in the large intestine.^35,43,44^

PHGG, made from the *Cyamopsis tetragonoloba* L seed is composed of a 1:2 ratio of galactose and mannose. The form commonly used in prebiotic powders is registered as Sunfiber.^44,45^ Doses ranging from 5 g to 10 g per day have been shown to reduce diarrhea frequency, improve stool consistency, modulate gut bacteria, and not interfere with nutrient absorption.^39,43,46,47^ One study of healthy controls with a tendency toward diarrhea stools demonstrated that supplementing with 5 g per day of PHGG for 3 months significantly improved stool consistency and increased *Bifidobacteria* populations.^46^ The same was seen in athletes prone to diarrhea distress, where supplementing with 6 g per day improved stool form, as well as significantly increasing populations of *Bifidobacteria* and other beneficial bacteria.^48^ Additional studies have shown that supplementing with PHGG after colorectal surgery can reduce stoma output and increase both butyrate producers and *Bifidobacteria*.^44,46,49^ At the same time, other studies have shown PHGG to reduce bloating and gas in individuals with IBS and to have a significant prophylactic effect on reducing diarrhea post-radiation therapy but not during active radiation treatment.^50,51^ Based on these findings, it was suggested that the patient take PHGG with a dose of 7.5 g daily pre, during and post chemo and radiotherapy treatment.

Lactulose, a non-digestible disaccharide of galactose and fructose, can increase motility at higher doses (>30 ml/day), but at lower doses (5–15 ml/day), can aid in improving beneficial bacteria populations, increase SCFA production, and acidify the colon.^52^ The prebiotic effects of lactulose promote the health of the gut microbiota by stimulating the growth of bifidobacteria while reducing levels of pathogenic bacteria, such as *Proteobacteria*.^52,53^ The patient was advised to take 15 ml daily of the prescribed lactulose by her physician based on the action of lactulose at a low dose. Note lactulose is a pharmaceutical medication in the United States. In other countries, lactulose is an over-the-counter drug.

Probiotics have been shown to reduce diarrhea, preserve the microbiota, and prevent the overgrowth of pathogenic bacteria during insults to the gut from medications such as chemotherapy and radiation.^11,28,53–56^ Some specific strains and multi-strain probiotics that have shown efficacy in reducing diarrhea, improving the diversity of the gut microbiota, and increasing colonic butyrate production include *Lactobacillus rhamnosus GG*, *Lactobacillus rhamnosus* HN001, *Lactobacillus acidophilus LAC-36, Lactobacillus acidophilus NCFM*, *Lactobacillus paracasei LPC-37*, *Bifidobacterium longum BB-536*, *Bifidobacterium lactis HN019*, Bifidobacterium lactis Bi-07, *Saccharomyces cerevisiae* var *boulardii* CNCM I-745, and Colon Dophilus™ and VSL#3 (multi-strain probiotics).^10,27,50,53–65^ *L. rhamnosus GG* has been shown to increase the rate of epithelial cell synthesis, healing gut barrier damage caused by chemotherapy and improving bacterial diversity while preventing the overgrowth of pathogenic bacteria.^56^ In a systematic review, *Saccharomyces cerevisiae* var *boulardii* CNCM I-745 was shown to be effective against antibiotic-associated diarrhea.^66^ *S. cerevisiae* var *boulardii* CNCM I-745 has antimicrobial and anti-inflammatory effects and increases the activity of brush border enzymes (lactase, alpha-glucosidase, alkaline phosphatase), which aids in the prevention of diarrhea and can prevent toxic damage from gut insults, such as that from *Clostridoides difficile* or *Escherichia coli* infections.^66,67^ The above mentioned strains are found in Florastor, Metagenics UltraFlora Acute, Metagenics UltraFlora IB and Culturelle (Table 2). For this reason, it was suggested that the patient follow the dose and timing as shown in Table 2. Based on the evidence and the observed gut microbiota changes as shown by 16S rRNA testing (Table 1), the selected strains of probiotics mentioned prior had some probable positive modulation to the gut microbiota.

This case report noted that a 57-year-old postmenopausal woman with BC undergoing chemotherapy and radiation therapy was able to achieve her goals of no diarrhea or constipation nor the return of her IBS symptoms throughout her cancer treatments. The 16S rRNA analysis showed the patient’s *Bifidobacterium* increased and *Proteobacteria* decreased compared to baseline. Butyrate producing genera had returned to baseline. The alpha-diversity was lower than at baseline (2.99% from 3.26%) but improved from March to August 2022 (2.58% to 2.99%). The authors propose that the patient’s goals were met due to the personalized approach using evidence-based prebiotics and probiotics. The patient’s adherence to a plant-focused Mediterranean diet and taking supplements could also have contributed to the positive outcome. However, based on other studies demonstrating the efficacy of prebiotics (PHGG and lactulose) and specific probiotics (such as *L. rhamnosus GG* and *S. cerevisiae* var *boulardii* CNCM I-745) in reducing diarrhea episodes, improving the mucosal lining and increasing microbiota diversity and balance, it is possible that the prebiotics and probiotics were one of the main contributors to preventing GI chemotherapy and radiation therapy adverse effects.

There are a few limitations to this case report. Multiple interventions were used throughout chemotherapy and radiation therapy, which makes it challenging to discern whether the intervention as a whole is needed to avoid adverse effects, or if the prebiotics and probiotics alone could elicit a positive change. The patient adhered to a Mediterranean-like diet before, during, and after chemotherapy and radiation therapies. The Mediterranean diet consists of high-fiber foods and polyphenols that can be beneficial to the health of the microbiota. Although more studies are needed on the direct effect of the Mediterranean diet on key species, it is possible that the patient’s diet contributed to positive changes in the microbiota.^68^ The study by Artale et al.^69^ illustrated a reduction in CID in patients with colorectal cancer who adhered to a Mediterranean diet. The study, however, did not measure the gut microbiota, making it difficult to know if the diet was associated with positive changes to the gut microbiota.

In addition, the patient took nutrient and nutraceutical supplementation before, during, and after chemotherapies and radiation therapies (Table 4). The supplementation stayed consistent other than previous chemotherapies. Therefore, it is unlikely that the supplements beneficially modulated the gut microbiota beyond that of the prebiotics and probiotics.

Additionally, before the diagnosis of BC, the patient had taken prebiotics and probiotics, and her bacteria diversity, butyrate-producing bacteria, and microbiota balance were not suboptimal. Another limitation is not testing the gut microbiome more regularly during treatment to monitor the shifts in butyrate-producing bacteria, populations of *Proteobacteria*, alpha-diversity, and *Bifidobacteria* more closely.

Lastly, the gut microbiota was tested using a commercially available 16s rRNA kit from Ombré Lab, and shipped to Laragen Sequencing & Genotyping and Eurofins Clinical Enterprise for processing. Therefore, the authors did not have direct oversight over the collection and processing of the samples.

## Conclusion

The use of specific prebiotics and probiotics for preventing CID and preserving gut bacteria balance has the potential to be a beneficial method for those undergoing breast cancer chemotherapy and radiation treatments. This case report is a narrative review of a personalized nutritional and lifestyle protocol that exhibited a possible effective non-pharmaceutical alternative to the standard of care for managing CID and GI issues associated with these interventions. The case report provides a potential hypothesis for further research to validate the safety and efficacy of prebiotics and probiotics in breast cancer and other cancer patients treated with chemotherapy and/or radiation therapies.

## Supplementary Material

Tables_and_Figures_Preventing_chemotherapy_induced_diarrhea_and_microbiota_imbalances_with_prebiotics_and_probiotics_in_breast_cancer_treatment_A_case_report_July_1_2024_submission-.docx

## Data access statement

The health data is stored in an EHR system to abide by HIPAA policies. The FASTQ files were submitted to the National Center for Biotechnology Information (NCBI). The accession number is SRP48933.
