# Supplementary material for: Preventing chemotherapy-induced diarrhea and microbiota imbalances with prebiotics and probiotics in breast cancer treatment: A case report
Source: Gut Microbes Rep. 2024 Aug 13;1(1):2379475. doi: 10.1080/29933935.2024.2379475 (PMC12940157; doi:10.1080/29933935.2024.2379475)

|  |  |  |
| --- | --- | --- |
|  |  | |

**Table 1**

*Key microbiota abundance and diversity*

|  | **Relative Abundance of Microbiota**ᵃ | | | **Date of 16S rRNA Sampling** | | |
| --- | --- | --- | --- | --- | --- | --- |
|  |  |  |  | |  |  |
|  |  | | | | | |
|  | Key Butyrate Producing Generaᵇ |  | April 27, 2021 | | March 10, 2022 | August 31, 2022 |
|  | *Faecalibacterium*  *Eubacterium (E. rectale only)*  *Roseburia*  *Anaerostipes*  *Clostridium*  *Ruminococcus*  *Coprococcus*  *Butyrivibrio*  *Butyricicoccus*  Total |  | 20.158%  0.779%  4.955%  2.369%  2.415%  1.464%  1.102%  0.007%  0.836%  34.085% | | 10.088%  0.259%  2.848%  0.613%  0.425%  0.593%  0.487%  0%  0.083%  15.396% | 15.797%  1.872%  9.326%  0.509%  0.867%  5.703%  0.502%  0.035%  0.066%  34.677% |
|  | Change compared to previous testᶜ |  |  | | -54.83% | 125.23% |
|  | *Proteobacteria*  Change compared to previous test |  | 1.604% | | 2.319%  44.58% | 0.521%  -77.53% |
|  |  | | | | | |
|  | *Bifidobacterium*  Change compared to previous test |  | 0.488% | | 2.719%  457.17% | 1.125%  -58.63% |
|  | Shannon alpha diversity index |  | 3.26% | | 2.58% | 2.99% |
|  | Change compared to previous test |  |  | | -20.86% | 15.89% |
|  |  | | | | | |
|  |  |  |  | |  |  |
|  |  |  |  | |  |  |
|  |  |  |  | |  |  |

ᵃ The data presented is a percentage of total genera abundance.

ᵇ Via the acetyl-CoA pathway

ᶜ Standard percentage change formula (((value 2 - value 1)/value 1) x 100)) was used to calculate the change from the previous test to the current.

**Table 2:** *Prebiotic and probiotic supplements*

|  | **Dose** | **Occasions administered** | |
| --- | --- | --- | --- |
|  |  | January 12-August 31, 2022 | Day prior to and  day of infusion* |
| **Probiotics** |  |  |  |
| 1. *Lactobacillus rhamnosus* | 10 billion CFU | Daily, a.m |  |
| GGᵃ |  |  |  |
|  |  |  |  |
| 2. *Saccharomyces cerevisiae var boulardii* | 250 mg | Daily, p.m. |  |
| CNCM 1-745 Biocodexᵇ |  |  |  |
|  |  |  |  |
| 3. A Probiotic Blendᶜ of:  *Bifidobacterium lactis* HN019 | 4 billion CFU (combined HN019 and HN001) |  | a.m., p.m |
| *Lactobacillus rhamnosus* HN001  *Saccharomyces cerevisiae var boulardii*  Unspecified strain | 5.5 billion CFU |  |  |
|  |  |  |  |
| 4. A Probiotic Blendᶜ of:  *Lactobacillus acidophilus*  NCFM | 60 billion CFU |  | a.m., p.m. |
| *Bifidobacterium lactis* |  |  |  |
| Bi-07 |  |  |  |
|  |  |  |  |
| **Prebiotics** |  |  |  |
| 1. Lactulose 10g/15mlᵈ, Rx (no additives) | 15ml | Daily, p.m. |  |
|  |  |  |  |
| 2. Partially Hydrolyzed Guar Gumᵉ (PHGG) | 7.5g | Daily, a.m. |  |
|  |  |  |  |

* March 30 and 31, 2022, May 1 and 2, 2022, May 22 and 23, 2022, June 12 and 13, 2022.

Supplement manufacturers:

ᵃ Amerifit (Cromwell, CT, USA)

ᵇ Biocodex (Beauvais, France)

ᶜ Metagenics (Aliso Viejo, CA, USA)

ᵈ Hi-Tech Pharmacal Co., Inc. (Amityville, NY, USA)

ᵉTaiyo International (Pittsburg, PA, USA)

**Table 3:** *Prebiotic and probiotic beneficial outcomes and informed-evidence*

| **Pre/Probiotic** | **Outcomes** | **Evidence** |
| --- | --- | --- |
| *Lactobacillus rhamnosus* GG | ↑ rate of epithelial cell synthesis    ↑ gut healing    ↑ bacterial diversity    ↓ pathogenic bacterial growth | Osterlund et al., 2007; Wei et al. 2018 |
| *Saccharomyces cerevisiae* var *boulardii* CNCM I-745 | ↓ antibiotic associated diarrhea    ↑ activity of brush border enzymes    ↓ pathogenic growth    + antimicrobial    + anti-inflammatory | Jahn et al., 1996; McFarland, 2010 |
| *Bifidobacterium lactis* HN019    *Lactobacillus rhamnosus* HN001    *Lactobacillus acidophilus* NCFM    *Bifidobacterium lactis* Bi-07 | ↓ bloating    ↓ diarrhea    ↓ pathogenic bacterial growth    ↑ bacterial diversity | Hemalatha et al. 2014; Ouwehand et al., 2014; Ringel–Kulka et al., 2011; Toscano et al., 2017 |
| Partially hydrolyzed guar gum (PHGG) | ↓ diarrhea post radiation    + stool consistency  ↓ bloating and gas    ↑ butyrate producing bacteria | Kapoor et al., 2020; Niv et al., 2016; Rosli et al., 2021; Yasukawa et al., 2019; |
| Lactulose | ↑ bifidobacteria    ↑ butyrate producing bacteria    ↓ *Proteobacteria*    + acidify the colon | Karakan et al., 2021; Maltz et al., 2020; |

**Table 4:** *Supplements*

| Supplements: | Dose | Supplements taken at diagnosis  Jan-Mar 2022 | Supplements taken during chemotherapyᵃ  Mar-Jun 2022 | Supplements taken during radiotherapyᵇ  Jul-Aug 2022 |
| --- | --- | --- | --- | --- |
| Vitamin d3  plus vitamin K | 125 mcg  20 mcg | ✓ | ✓ | ✓ |
| Multivitaminᶜ | 1 cap daily | ✓ | ✓ | ✓ |
| Vitamin B6 | 50 mg |  | ✓ | ✓ |
| Camellia sinensis  (Green tea extract) | 200 mg | ✓ | ✓ | ✓ |
| Zingiber officinale  (Ginger) | 500 mg |  | ✓ |  |
| Magnesium | 240 mg | ✓ | ✓ | ✓ |
| Melatonin | 3 mg | ✓ | ✓ | ✓ |
| EPA and  DHA | 600 mg  400 mg | ✓ | ✓ | ✓ |
| Probiotics | See table 2 | ✓ | ✓ | ✓ |
| Prebiotics | See table 2 | ✓ | ✓ | ✓ |
| Vitamin B12 | 2000 mcg | ✓ | ✓ | ✓ |
| Vitamin E  (mixed tocopherols) | 400 mg | ✓ | ✓ |  |
| Curcuma longa  (Curcumin) | 500 mg | ✓ |  | ✓ |
| Trametes versicolor   (Turkey Tail mushroom) | 500 mg | ✓ |  | ✓ |
| Iodine | 225 mcg | ✓ |  |  |
| CoQ-10 | 60 mg | ✓ |  |  |
| Calcium D-glucarate | 500 mg | ✓ |  |  |

ᵃ Supplements approved by pharmD & integrative oncologist.

ᵇ Supplements approved by radiation oncology team.

ᶜ Pure Encapsulations O.N.E. Multivitamin.

✓ =  supplements taken.

**Table 5**

*Medications*

| Medications:  Pre-diagnosis and throughout treatment | Antibiotic administered at each surgery:  Jan 22, 2022  Feb 11, 2022  Feb 25, 2022 | Chemotherapy  1 cycle:  March 31, 2022 | Chemotherapy  3 cycles:  May 2, 2022  May 23, 2022  June 13, 2022 | Chemotherapy support medications*: (steroids, anti-nausea, pain)  March 31, 2022- June 13, 2022 |
| --- | --- | --- | --- | --- |
| Levothyroxine 88mcg/daily | ceFAZolin (ANCEF) IVPB | cyclophosphamide (CYTOXAN) IV | cyclophosphamide (CYTOXAN) IV | fosaprepitant (EMEND) |
|  |  | DOXOrubicin 2 (ADRIAMYCIN) IV PUSH | DOCEtaxel (TAXOTERE) IV | dexamethasone (DECADRON) |
|  |  | pegfilgrastim subcutaneous | pegfilgrastim subcutaneous | heparin lock  (HEP-LOCK) |
|  |  |  |  | NaCl |
|  |  |  |  | ondansetron (ZOFRAN) |
|  |  |  |  | OLANZapine (ZyPREXA) |
|  |  |  |  | prochlorperazine |
|  |  |  |  | acetaminophen |
|  |  |  |  | oxyCODONE (ROXICODONE) |
|  |  |  |  | loratadine |

* Some chemotherapy support medications were “only as needed”.

**Table 6**: *Toxicity assessments and diarrhea grading*

| **Toxicity Screening Dates** | | | | | |
| --- | --- | --- | --- | --- | --- |
|  | May 2, 2022 | May 9, 2022 | June 2, 2022 | June 13, 2022 | June 20, 2022 |
| **Adverse Event (AE) Diarrhea*** |  |  |  |  |  |
| Grade 1:  increase of <4 stools/day over pre-treatment | ✓ | ✓ | ✓ | ✓ | ✓ |
|  |  |  |  |  |  |
| Grade 2:  increase of 4-6 stools/day, or nocturnal stools | - | - | - | - | - |
|  |  |  |  |  |  |
| Grade 3:  increase of >/=7 stools/day or incontinence; or needs for parenteral support for dehydration | - | - | - | - | - |
|  |  |  |  |  |  |
| Grade 4:  physiologic consequences requiring intensive care; or hemodynamic collapse | - | - | - | - | - |
|  |  |  |  |  |  |
| Grade 5:  Death related to adverse event | - | - | - | - | - |

* Common Terminology Criteria for Adverse Events (CTCAE) definition of diarrhea: A disorder characterized by an increase in frequency and/or loose or watery bowel movements (National Cancer Institute, 2017).

✓ = Patient experience.

**Figure 1:** Case timeline


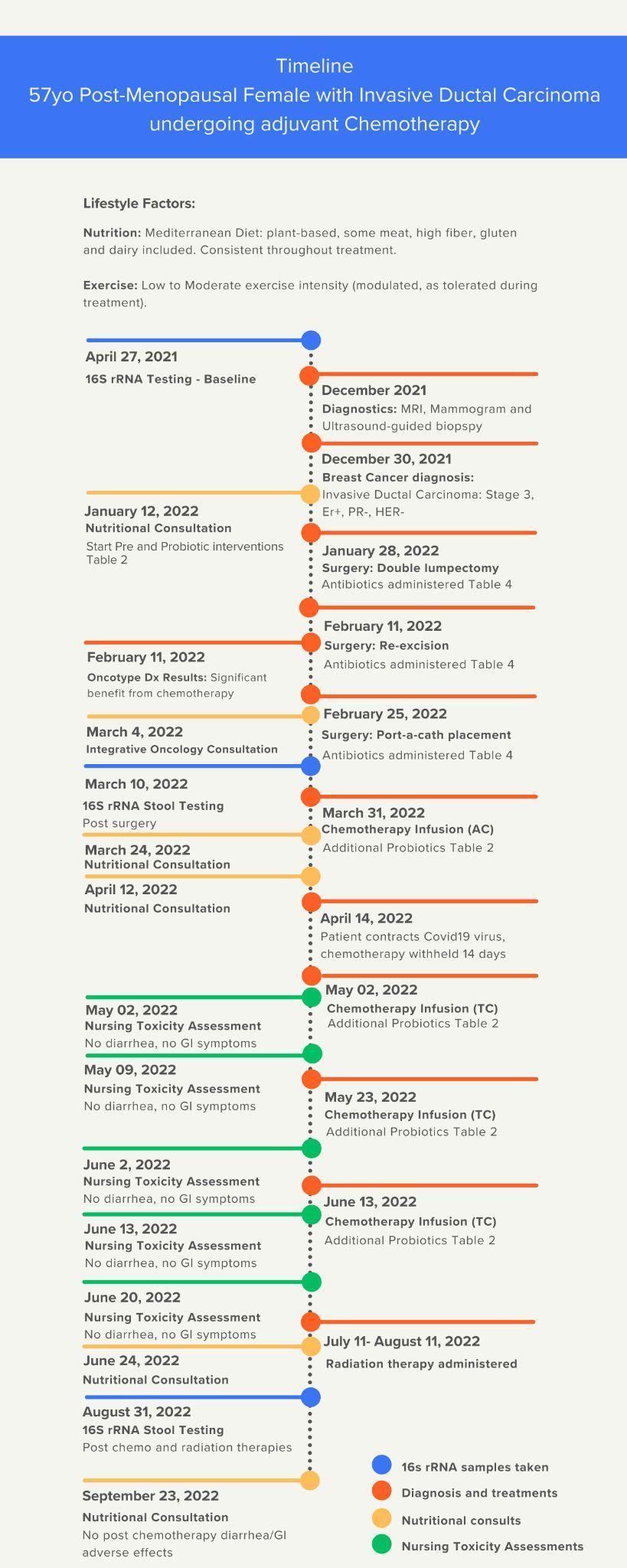


**Figure 2:** Most abundant genera by date of sample collection


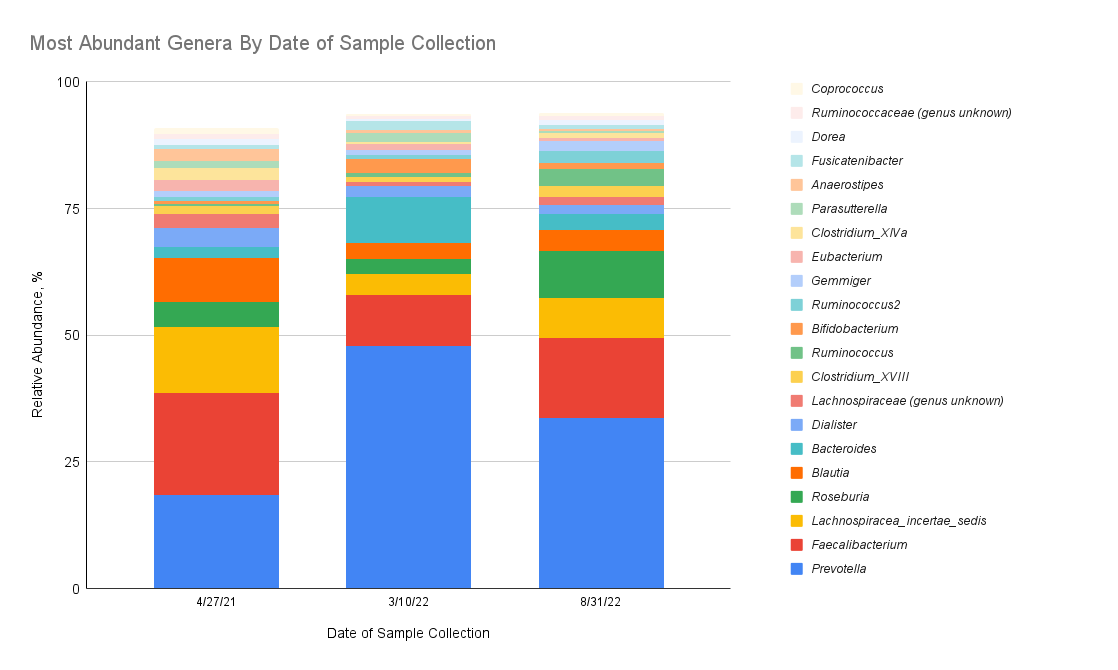

Supplement: Tables_and_Figures_Preventing_chemotherapy_induced_diarrhea_and_microbiota_imbalances_with_prebiotics_and_probiotics_in_breast_cancer_treatment_A_case_report_July_1_2024_submission-.docx [file KGMR_A_2379475_SM2841.docx]
